# Mediating Effect of Challenges on Demographics and Coping Strategies of Indian Healthcare Workers during COVID-19

**DOI:** 10.3390/ijerph20054474

**Published:** 2023-03-02

**Authors:** Anahita Ali, Santosh Kumar

**Affiliations:** Faculty of Public Health, Poornima University, Jaipur 303905, India

**Keywords:** mediation model, coping strategies, Indian healthcare workers, COVID-19, mental health

## Abstract

Healthcare workers (HCWs) including doctors, nurses and allied workers struggled to cope up with the stressful situation as the COVID-19 pandemic unsettled healthcare systems, including India’s. Many factors (commonly called as stressors) acted as major sources of stress and resulted in poor mental health of HCWs. Therefore, this study predicted and explained the mediating effect of challenges on demographic characteristics and coping strategies of HCWs. Data from a cross-sectional study was collected from the district hospital of Rajasthan, India, during the period of August 2022–October 2022. HCW’s experience level, shift type and distance of greenspaces from their accommodation were significantly correlated with the challenges they faced at work, specifically societal challenges. Thus, HCWs were more inclined to adopt a meaning-focused coping strategy to retain good mental health during the pandemic. Therefore, these findings call for interventions requiring a layered response, comprising strategies and actions that are structural. At the organizational level, these actions may provide supportive workplace environments.

## 1. Introduction

The unpredicted COVID-19 pandemic had a major adverse impact on the overall public healthcare system globally, including in India. The sudden increase in COVID-19-positive patients, associated severity, infrastructural crisis and overburdened healthcare facilities across the countries resulted in a healthcare crisis never seen before [1]. Healthcare workers (HCWs) including doctors, nurses and allied workers struggled to cope up with the stressful situation as the second wave unsettled the healthcare systems. It resulted in unfavorable circumstances due to stretched resources, shortage of healthcare workforce, inadequate protective equipment supply, limited availability of healthcare facilities, increased risk of contracting the COVID-19 virus and associated risk of spreading the infection to family and others. These factors (commonly called stressors) acted as major sources of stress and resulted in poor mental health of HCWs.

Since the pandemic, HCWs have developed negative emotions and feelings such as anger, irritation and fear [2]. The prevalence of these negative emotions was commonly reported in previous studies in which HCWs typically reported grief and anger as their primary negative emotions [2,3,4]. However, the negative emotional state of HCWs was changed during the first and second waves, indicating that negative emotions and feelings were higher than usual and gradually increased over time. These findings suggest that the COVID-19 pandemic’s detrimental effects on HCWs’ mental health, including emotional health, endure over time. According to previous research, one of the major causes of poor mental health is an imbalance in healthcare service demand and supply. These include healthcare service availability, infrastructure, and human resources in both the public and private sector [3]. Unfortunately, it was difficult for the collectives to bridge this gap while also meeting the requirements of a population from various socioeconomic groups. The integration of these factors contributes to the challenges and associated stresses being experienced by HCWs. More commonly, HCWs reported using emotion-focused coping strategies to regulate the negative emotions developed from stressful situations such as the COVID-19 pandemic and other stressors (such as organizational challenges). Emotion-focused coping is helpful in regulating negative emotions rather than controlling the stressful situation. It includes watching TV, reading books, behavioral disengagement and many others. These coping strategies help reduce symptoms associated with poor mental health [2,5,6].

HCWs working in different shifts are more vulnerable to poor mental health, including poor sleep quality and diminished sleep during public crises such as the COVID-19 pandemic [1]. For instance, night-shift HCWs in Paris public hospitals had poor quality of working life (QWL) as the effects on them were underestimated during the COVID-19 pandemic. It negatively affected their relationships with colleagues and acted as a source of tension [7]. The consequences of poor mental health include reduced work performance, worse mood, physical and mental tiredness at work. Additionally, the current COVID-19 pandemic has added to the workload of HCWs—a stressor for poor mental health. Shift workers became sleep deprived during the COVID-19 pandemic; a significant association between night shifts and diminished sleep was reported in HCWs posted in COVID-19 wards rather than regular medical wards [8,9,10].

To cope with poor mental health developed from poor sleep quality and altered working environments, adopting a problem-focused coping strategy is beneficial for HCWs because it is positively associated with well-being and good sleep [11]. It refers to proactive measures taken to deal with stressful events and change a problematic person–environment interaction in order to reduce or eliminate the causes of stress through personal conduct. For instance, encouraging the HCWs to create to-do list, improving problem-solving skills, establishing healthy boundaries, asking for support from professionals and friends, and time management skills.

Apart from regulating the negative emotions through emotion-focused coping and improving sleep quality through problem-focused coping, green spaces contribute to good mental health. The term “green space” refers to green areas such as paved green areas, open and enclosed burial sites, sports fields, private green spaces, informal and formal green forests, road medians, vacant lots, and horticulture [12]. Previous research has well documented the positive effects of green spaces on mental health. In quality-of-life models, urban green areas are credited with promoting physical comfort, psychological and emotional relaxation, and social engagement. Green spaces help reduce stress levels, encourage social contact, and promote good mental health. Unfortunately, inaccessibility of green spaces during crises such as the COVID-19 pandemic may act as a source of stress for HCWs [13,14,15]. Therefore, access to green spaces is important because it enables people to connect with nature—a type of meaning-focused coping strategy. Using this coping strategy, people use introspective goals (e.g., an intention in life or core values) and beliefs (e.g., spiritual, beliefs about justice or religion) for good mental well-being.

Finally, lack of social support is another source of stress among HCWs during the COVID-19 pandemic as reported in previous studies [16]. For instance, Chinese HCWs in the early stages of the COVID-19 pandemic reported a significant correlation between social support and their mental health [17,18]. Similarly, HCWs who visited the elderly for home visits during the pandemic had a positive correlation with social support seeking and mental health [19]. It is likely that social support may reduce stress among HCWs during the COVID-19 pandemic because previous studies have reported an association between stress and mental health mediated by social support [17].

Previous research studies have reported the significant correlation between poor mental health and challenges (called stressors) such as poor sleep, lack of social support, inadequate infrastructure, shift time and many others faced by the HCWs globally. However, there is limited literature that reports the mediating effects of challenges faced by Indian HCWs on their coping behavior and demographic factors. Unfortunately, there is a scarcity of literature to fully address this existing gap. In this line, we aimed to predict and explain the mediating effect of challenges faced by Indian HCWs on their demographic characteristics and coping behavior. We considered Indian HCWs’ challenges as a mediating variable to examine whether less or more challenges have an effect on demographics and coping strategy adoption by HCWs. To test the mediating effect of challenges, the postulated hypotheses for Proposed Model are mentioned as following.

**Hypothesis 1a:**  *Education of HCWs influences emotion-focused coping*.

**Hypothesis 1b:**  *Designation of HCWs influences emotion-focused coping*.

**Hypothesis 1c:**  *Experience of HCWs influences emotion-focused coping*.

**Hypothesis 1d:**  *Shift type (day or night duty) of HCWs influences emotion-focused coping*.

**Hypothesis 1e:**  *Distance of green spaces from accommodation influences emotion-focused*.

**Hypothesis 2a:**  *Education of HCWs influences problem-focused coping*.

**Hypothesis 2b:**  *Designation of HCWs influences problem-focused coping*.

**Hypothesis 2c:**  *Experience of HCWs influences problem-focused coping*.

**Hypothesis 2d:**  *Shift type (day or night duty) of HCWs influences problem-focused coping*.

**Hypothesis 2e:**  *Distance of green spaces from accommodation influences problem-focused coping*.

**Hypothesis 3a:**  *Education of HCWs influences meaning-focused coping*.

**Hypothesis 3b:**  *Designation of HCWs influences meaning-focused coping*.

**Hypothesis 3c:**  *Experience of HCWs influences meaning-focused coping*.

**Hypothesis 3d:**  *Shift type (day or night duty) of HCWs influences meaning-focused coping*.

**Hypothesis 3e:**  *Distance of green spaces from accommodation influences meaning-focused coping*.

## 2. Materials and Methods

### 2.1. Study Site and Design

India is considered a biotechnological and pharmaceutical hub in the world. Unfortunately, with increasing population (India recently surpassed China’s population and became the most populous country in the world, having a population of 1.4 billion), it fails to provide equitable and adequate healthcare services in most of its regions, particularly rural areas of the country. The existing HCW-to-population ratio is 21 per 10,000 indicating a shortage of healthcare professionals in India. The country needs a minimum of 1.8 million HCWs (doctors, nurses and midwives) to achieve a minimum threshold (45 per 10,000 by 2030) and close the existing gap [20]. As of 2019, there were 3.87 million (67.2%) HCWs (doctors, nurses, dentists, pharmacists, traditional medicine practitioners) of which 2.48 (43%) were adequately qualified [21].

Rajasthan, a north-western state of India, accounts for 5% of the country’s population (approximately 82.5 million) and has the largest geographical area in India. It has six zones and 33 districts with every district providing primary, secondary and tertiary care. There are dedicated district health care facilities (both government and private) in every district. A few districts have extended healthcare facilities—satellite hospitals—to improve the access to healthcare services for those living in remote areas, away from the city center. Rajasthan has 4% of doctors and 10.3% of nurses out of the country’s total healthcare workforce. There are approximately 3000 HCWs employed in the target district hospital which was considered for this study.

A cross-sectional study design was used to collect data from a government district hospital in Rajasthan, India, during the period of August 2022–October 2022.

### 2.2. Participants and Procedures

The study population considered for the study were HCWs currently employed in the district hospital, which included doctors, nurses, administration staff, and allied workers. The total number of participants in the sample obtained for the study was 764, of which 245 were doctors, 307 were nurses, 194 were allied workers and 18 were administration staff. As there was a small group of administrative staff, we included all of them. To determine the mentioned sample size for the study, Cochran’s formula was used, at 95% confidence interval, 5% acceptable margin of error, and 20%, 28% and 15% was estimated as proportions of the problem for doctors, nurses, and allied workers, respectively [22]. After considering a 10% non-response rate, we contacted 840 HCWs. A total of 83 HCWs did not respond and the final sample for this study was 759.

We included all the doctors, nurses, allied workers, and administration staff working in the hospital. Nonmedical staff such as security guards, receptionists and other employees were excluded. All those who did not give their consent for participation and/or could not be approached after three attempts were also excluded. We used a simple random sampling method to select participants after assigning a unique number. A list of participants was created from which the participants were selected randomly.

### 2.3. Data Collection Tool

In order to evaluate the coping strategies of the respondents, the Brief–COPE Inventory was used in the study, which is a condensed version of Carver’s COPE scale [23]. The data was collected with the help of a Likert scale—a four-point scale where 1 represented “I haven’t been doing this at all”, and 4 represented “I have been doing this a lot”.

### 2.4. Statistical Analysis

To analyze the data, we performed descriptive statistics by using Epi-info software version 7. The challenges were compared with various demographic characteristics of the participants with the help of Chi-square test and Fisher’s exact test, where appropriate. Similarly, to obtain the statistical association between coping strategies and demographic characteristics of the respondents, Chi-square test and Fisher exact’s test, where appropriate, were used. A *p*-value < 0.05 was considered to demonstrate statistical significance of the variables.

The hypothesized mediation model was tested in a single model using a bootstrap approach to assess the significance of indirect effect at differing levels of mediator. Demographic characteristics were the predictor variable and challenges faced at different levels (individual, organizational and societal) as mediators. The outcome variable was the coping strategies adopted by the participants. RMediation package version 10.6-1 was used with bias-corrected 95% confidence interval.

**Figure 1:**
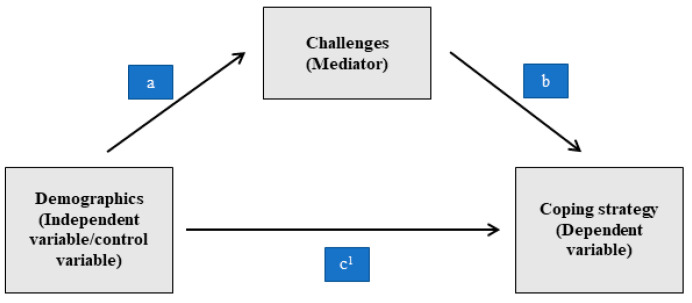
Proposed mediation path to explain association between demographics of healthcare worker, challenges faced by them and their coping response.

The initial path model (Figure 1) represents the relationship between constructs that influence the coping strategy adoption (emotion focused, problem focused, meaning focused) and demographic characteristics (age, gender, marital status, education, designation, experience level, shift type and distance and frequency of visiting green spaces from accommodation).

Path “a” represents the causal relationship between the HCWs’ demographic characteristics and challenges faced by them at different levels. It indicates that demographic factors such as being female, senior designation and high experience level increased the number of challenges faced at the workplace during the COVID-19 pandemic. Path “b” represents the causal relationship between HCWs’ challenges and their coping behavior such as emotion-focused, problem-focused, or meaning-focused coping strategy. It indicates that a higher number of challenges resulted in greater coping strategy adoption by HCWs. Finally, path “c^1^” represents the causal relationship between the HCWs’ demographics and coping behavior, mediated by the challenges faced by them. It indicates that having a higher educational degree, working at a senior level, poor accessibility of green spaces and many others demographic factors increased the number of challenges faced by them in the workplace and in turn, increased their coping strategy adoption during the COVID-19 pandemic.

Based on the proposed framework, we assumed that the challenges faced by the HCWs would mediate the effect of their demographic characteristics on coping strategy adoption. The indirect effects were tested to assess whether challenges mediate the relationship. We considered *p*-value < 0.01 as significant path in the proposed model.

### 2.5. Ethics Approval of Research

The study was approved by our university’s institutional review board IRB (JSPH-IRB/2022/06/26) and our hospital’s ethics committee (SNMC/IEC/2022/618). The study’s purpose, associated risks, benefits and importance of their participation was explained to the participants, after which they gave their written informed consent for voluntary participation. They were also informed about the confidentiality and non-disclosure of their personal identities. All the procedures were followed by maintaining the ethical standards as outlined in the Declaration of Helsinki.

## 3. Results

### 3.1. Demographic Characteristics of the Participants

Out of the total 759 participants, 270 (36%) were doctors, 325 (43%) were nurses, 146 (19%) were allied workers, and 18 (2%) were administration staff. In total, 384 (51%) were female and 375 (49%) were male participants. The mean age was 35 years and 38 years among females and males, respectively. Of the total, 268 (35%) participants had a graduate degree, 286 (38%) participants had a diploma or polytechnic degree, and 197 (26%) participants had a postgraduate degree. Furthermore, 562 (74%) of the participants were married and 179 (24%) were unmarried. A majority (n = 450, 59%) of the participants had 1–5 years of experience in the current hospital, 157 (21%) had 5–10 years of experience and 152 (20%) had more than 10 years of experience. With respect to working pattern, 476 (63%) had day time shifts, 74 (10%) had night shifts and 209 (27%) had rotation shifts. As concerns green spaces, 285 (38%) respondents had green space within 0.5 km of their accommodation, 234 (31%) had green spaces 0.5–2 km away and 240 (32%) had more than 2 km distance from their accommodation to green spaces.

### 3.2. Correlation between Demographics, Challenges and Coping Strategies

**Figure 2:**
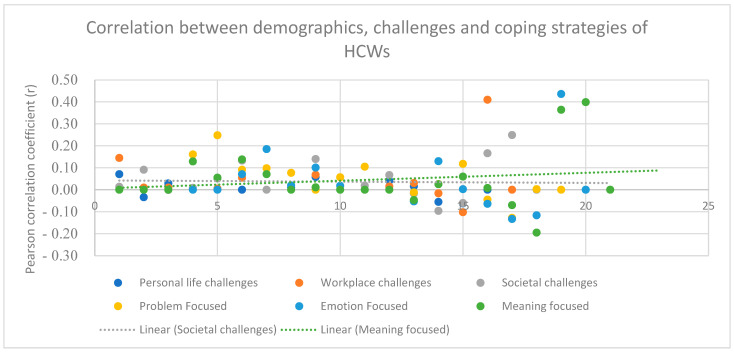
Scatter graph of correlation between demographic variables, challenges and coping strategies of HCWs.

The commonly reported coping strategies—emotion focused, problem focused and meaning focused—showed significant correlation with education level, experience level, shift type, distance of green spaces from HCWs’ accommodation, workplace challenges and societal challenges (*p*-value < 0.01, r = −0.06, 0.40, −0.12, 0.04, 0.14 and 0.01, respectively) (Figure 2).

The negative significant correlation (*p*-value < 0.01, r = −0.06 and −0.12) indicated that lower education levels (such as having diploma and graduate degree) and shift timings during the COVID-19 pandemic did not act as a challenge for HCWs and, in turn, they did not adopt a coping strategy.

The positive significant correlation (*p*-value < 0.01, r = 0.40, 0.04, 0.14 and 0.01) indicated that higher experience level (which implies having more roles and responsibilities in the hospital), larger distance to green spaces from their accommodation (implying poor accessibility), shortage of personal protective equipment, burnout, anxiety, depressive symptoms, poor sleep quality, improper guidelines on patient handling and fear of alienation from the society (due to myths related to the COVID-19 pandemic) resulted in poor mental health and in turn, the HCWs adopted more coping strategies to maintain their good mental health.

**Figure 3:**
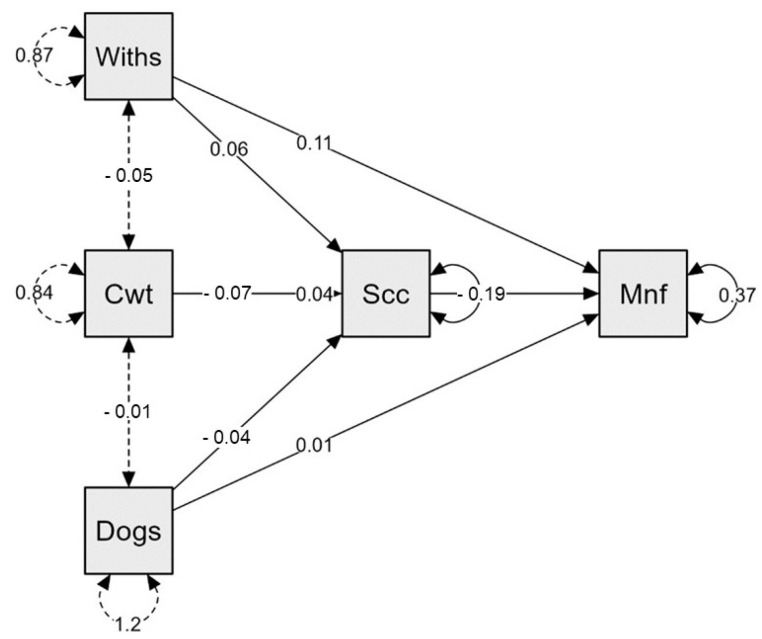
Path analysis model for the mediating effect of challenges on HCWs’ demographics and coping strategy adoption during the COVID-19 pandemic. Note: Withs = Working in this hospital (experience), Cwt = Current working type, Dogs = Distance to green spaces from accommodation, Scc = Societal challenges, Mnf = Meaning focused.

The mediation model in Figure 3 was obtained after multiple fitness evaluation with model of fitness with insignificant variable exclusion. The model was reduced to seven paths and was evaluated for goodness of fit. In this model, experience level of HCWs, shift type (Cwt) and distance to green spaces from their accommodation (Dogs) were antecedents (predictors) to challenges faced by them (specifically societal challenges—Scc) while meaning-focused coping (Mnf) strategy was the dependent variable.

The statistically significant correlation (*p* < 0.01, r = 0.11) between experience level of HCWs (Withs) with meaning focused coping (Mnf) indicated that greater experience of the HCWs such as senior staff adopted more meaning focused coping strategy. For instance, recreational activities and religious activities to manage their mental health during COVID-19 pandemic. A similar statistically significant correlation (*p* < 0.01, r = 0.06) with societal challenges (Scc) indicated that senior HCWs faced more challenges at the workplace (specifically societal challenges due to myths and misinformation about COVID-19; thus, they faced more alienation, discrimination from society and hard time while managing patients and their families in the hospital).

HCWs’ shift type (Cwt; day, night or rotation shift) had a statistically significant correlation (*p* < 0.01, r = −0.07) with societal challenges (Scc) faced during the COVID-19 pandemic. As reported by HCWs, they had alternative shifts in which they used to stay at home for seven days and work for the next seven days. Many HCWs also worked alternative days and some became COVID-19 positive after which they stayed quarantined in their houses for up to fourteen days. The inverse relationship between shift type and social challenges indicated that the changing shift schedules of HCWs negatively affected their relationship with their lessor (because many of them were living in rented houses) or neighbors. They faced non-cooperation from their lessor and they were asked to vacate the rented place. This behavior was seen due to the fear of COVID-19 infection transmission to the lessors, their family and neighbors.

Finally, societal challenges (Scc) had a statistically significant correlation (*p* < 0.01, r = −0.19) with meaning-focused coping (Mnf) behavior of HCWs during the COVID-19 pandemic. This inverse relationship indicated that those who faced more challenges from society such as alienation, denial and discrimination adopted fewer meaning-focused coping strategies to cope with the stressful situation. This could be due to limited or no accessibility to green spaces and other recreational facilities, resulting in them staying indoors in isolation during the COVID-19 pandemic. This may have affected their social relationships as they stayed away from social gatherings.

### 3.3. Parameter Estimates

The parameter estimates were conducted for direct and indirect effects of education, designation, experience level, shift type, and distance of green spaces on challenges (personal, organizational and societal) and coping strategies (emotion focused, problem focused and meaning focused) (Table 1).

#### 3.3.1. Direct Effects

The regression analysis of the demographics with emotion-focused coping strategy showed statistically significant direct effects of shift type and distance to green spaces (*p* < 0.001). Education, experience level and shift type showed statistically significant direct effects on problem-focused coping strategy (*p* < 0.001). Finally, designation and experience levels showed statistically significant direct effects on meaning-focused coping strategy (*p* < 0.001).

#### 3.3.2. Indirect Effects

No observable significant indirect effects of the shift type and distance to green spaces on emotion-focused coping strategy were obtained. Education, experience level and shift type showed no statistically significant indirect effects on problem-focused coping strategy. However, experience level, shift type and distance to green spaces showed significant indirect effects on meaning-focused coping strategy (*p* < 0.001) (Table 2).

#### 3.3.3. Total Effects

The shift type and distance to green spaces showed statistically significant total effect on emotion-focused coping strategy (*p* < 0.001). Education, designation and shift type showed statistically significant total effect on problem-focused coping strategy (*p* < 0.001). Only experience level had statistically significant direct total effects on meaning-focused coping strategy (*p* < 0.001) (Table 3).

Therefore, of 15 hypotheses, only 7 hypotheses showed significant association and were supported in the mediation model. We accepted the following hypotheses:

**Hypothesis 1a:**  *“Education of HCWs influences emotion-focused coping adoption”*.

**Hypothesis 1b:**  *“Designation of HCWs influences emotion-focused coping adoption”*.

**Hypothesis 2a:**  *“Education of HCWs influences problem-focused coping adoption”*.

**Hypothesis 2b:**  *“Designation of HCWs influences problem-focused coping adoption”*.

**Hypothesis 2c:**  *“Current working/shift time of HCWs influences problem-focused coping adoption”*.

**Hypothesis 3b:**  *“Designation of HCWs influences meaning-focused coping adoption”*.

**Hypothesis 3c:**  *“Experience of HCWs influences meaning-focused coping adoption”*.

## 4. Discussion

The current study reported that HCWs’ experience level, shift type and distance to green spaces from their accommodation were significantly correlated with the challenges they faced at work, specifically societal challenges and their inclination to adopt a meaning-focused coping strategy to retain good mental health during the pandemic.

Anger, grief, and fear were commonly experienced by HCWs throughout the pandemic [2]. A similar finding was reported in the current study as the HCWs felt emotionally tired and stressed for most of the time during the first and second wave of the COVID-19 pandemic. The findings are in line with those from Palestine who reported high levels of stress [24]. Increased stress levels among HCWs were a major factor in seeking social support [16]. In the present study, a majority of the participants faced various challenges, of which half of them reported fear from society due to alienation (societal challenge). Similar societal challenges were reported by the Indian HCWs who worked in slum areas during the COVID-19 pandemic and faced difficulties due to stigma among the slum population [25].

Research suggests that coping strategies and social support are significantly correlated with better mental health [26]. A similar correlation was obtained in the present study. These findings are line with those regarding women workers from service sectors who reported similar positive correlation between coping strategies during the COVID-19 pandemic [19]. Similarly, stress faced by the nurses from the oncology department had a positive correlation with their coping behavior [27]. As a workplace challenge, a significant effect on the stress and emotion-focused coping among academics in Ireland was reported [28]. However, the findings of the present study are in contrast as there was no mediating effect of workplace challenges on coping behavior. In the current study, shift type such as night or day duty was found to be significantly correlated with problem-focused coping. These findings are contrary to those from China in which negative coping behavior moderated the work pressure, work load and work time of nurses [8].

Perceived challenges faced at work may result in burnout among medical professionals. These perceptions may also influence their coping behavior [29]. In this line, the present study reported contrasting results as the workplace challenges did not completely mediate the coping behavior path, rather added to the stress in the work/life routine of HCWs [30]. Increased levels of anxiety and depressive symptoms were reported by HCWs due to stressful and unpleasant work environments during the COVID-19 pandemic [31]. Similar symptoms were also reported by Iranian nurses which led to a loss of peace and imbalanced personal life due to limited contact with family members [32]. Similarly, the uncertainty and last-minute work schedules severely impacted the daily lives of those working in Denmark’s hospitals [33]. Another indirect consequence is HCWs’ weariness in the form of tiredness and irritability [34,35]. Those working in Iran experienced similar physical tiredness, as well as skin damage and hormonal imbalance [32]. These findings are line with the current study in which HCWs reported physical tiredness, burnout, exhaustion, improper guidelines in the hospital and shortage of personal protective equipment.

However, young healthcare professionals and those working in clinical departments have higher stress levels, anxiety, and depression than their senior coworkers and those in non-clinical departments [31,35]. Conversely, there was a positive correlation obtained between the designation of HCWs and their coping behavior in the present study. It indicated that senior HCWs who generally have higher roles and responsibilities may have adequately coped with the stress. Additionally, sleep deprivation was associated with a lack of personal protective equipment at work and higher anxiety levels [36,37]. The primary reasons for greater levels of stress and anxiety among healthcare personnel were stigma, discrimination, and being overburdened with work [38]. These findings are in line with the present study as Indian HCWs feared societal discrimination, boycott and alienation. These societal challenges led to poor mental health.

As people may have limited green space within their homes, during the COVID-19 pandemic it was recommended to address green space accessibility for people. Unfortunately, during the COVID-19 pandemic, public parks and green spaces were closed. Maintaining appropriate access to green spaces could counteract the negative effects of crisis [15,39]. In line with the previous studies, the present study reported significant association between the accessibility of green spaces and their smaller distance from the homes of HCWs. These findings indicated that greater accessibility to green spaces enable HCWs to cope with the stressful situation. Poor or no accessibility to green spaces for recreational activities may lead to poor adoption of coping strategies resulting in poor mental health.

## 5. Conclusions

Based on the findings from the current study it can be stated that Indian HCWs fighting the COVID-19 pandemic felt burned out, irritated and stressed. It is suggested that specific interventions to address HCWs’ workplace challenges should be implemented to help them better cope with the crisis, enhance their mental health at work, and prevent mental health-related problems in the ongoing COVID-19 pandemic. In general, we accepted our assumptions that working at senior level, higher education level and more experience among HCWs are significantly correlated with increased coping strategy adoption specific to emotional regulation and problem management during stressful situations such as the COVID-19 pandemic to maintain good mental health. However, this study does not indicate an effective and active coping strategy adoption by HCWs. Therefore, these findings call for interventions requiring a layered response, comprising strategies and actions that are structural. At an organizational level, these actions may provide supportive workplace environments.

This study was conducted to predict the challenges faced by Indian HCWs that influence the causal relationship between their demographic characteristics and coping behavior. It supports the previous literature that workplace challenges negatively impacted the mental health of HCWs during the COVID-19 pandemic. However, as limitations: Firstly, the study participants from different departments were considered as a single sample instead of sub-dividing them into their respective departments. Therefore, this study may not have predicted the effects of challenges faced by Indian HCWs from different hospital departments. Secondly, the causal relationships between the variables may not have been reported due to the cross-sectional study design. Finally, we included the challenges faced by HCWs to test their mediating effects. However, factors other than challenges that were not considered in the present study may also have influenced their coping behavior.

## Figures and Tables

**Table 1:** Direct effects of demographics on challenges and coping strategy.

Direct Effects								
							95% Confidence Interval	
			Estimate	Std. Error	z-Value	*p*	Lower	Upper
Education	→	Emotion Focused	−0.003	0.022	−0.124	0.902	−0.049	0.047
Current designation	→	Emotion Focused	0.039	0.024	1.610	0.107	−0.021	0.098
Working in this hospital since	→	Emotion Focused	0.055	0.019	2.951	0.003	0.018	0.090
Current working/shift time	→	Emotion Focused	0.105	0.020	5.215	<0.001	0.067	0.145
Distance to green spaces	→	Emotion Focused	0.059	0.016	3.744	<0.001	0.024	0.089
Education	→	Problem Focused	0.291	0.043	6.706	<0.001	0.214	0.380
Current designation	→	Problem Focused	0.178	0.048	3.710	<0.001	0.089	0.276
Working in this hospital since	→	Problem Focused	0.120	0.037	3.251	0.001	0.032	0.193
Current working/shift time	→	Problem Focused	0.197	0.039	5.025	<0.001	0.124	0.271
Distance to green spaces	→	Problem Focused	0.062	0.031	2.037	0.042	−0.006	0.126
Education	→	Meaning focused	0.028	0.028	1.017	0.309	−0.030	0.081
Current designation	→	Meaning focused	0.122	0.031	3.977	<0.001	0.060	0.192
Working in this hospital since	→	Meaning focused	0.121	0.024	5.140	<0.001	0.071	0.166
Current working/shift time	→	Meaning focused	0.069	0.025	2.754	0.006	0.019	0.117
Distance to green spaces	→	Meaning focused	0.017	0.020	0.884	0.377	−0.020	0.057

*Note.* Delta method standard errors, bias-corrected percentile bootstrap confidence intervals, ML estimator.

**Table 2:** Indirect effects of demographics on challenges and coping strategy.

Indirect Effects										
									95% Confidence Interval	
					Estimate	Std. Error	z-Value	*p*	Lower	Upper
Education	→	Personal life challenges	→	Emotion Focused	2.755 × 10^−4^	0.001	0.263	0.793	−0.003	0.009
Education	→	Workplace challenges	→	Emotion Focused	−3.845 × 10^−4^	0.003	−0.153	0.878	−0.005	0.005
Education	→	Societal challenges	→	Emotion Focused	−0.001	0.002	−0.795	0.426	−0.007	0.001
Current designation	→	Personal life challenges	→	Emotion Focused	−1.649 × 10^−4^	6.648 × 10^−4^	−0.248	0.804	−0.005	0.002
Current designation	→	Workplace challenges	→	Emotion Focused	0.004	0.003	1.157	0.247	1.995 × 10^−6^	0.012
Current designation	→	Societal challenges	→	Emotion Focused	0.002	0.002	0.996	0.319	−6.163 × 10^−4^	0.008
Working in this hospital since	→	Personal life challenges	→	Emotion Focused	−2.131 × 10^−4^	8.140 × 10^−4^	−0.262	0.793	−0.006	0.002
Working in this hospital since	→	Workplace challenges	→	Emotion Focused	−0.003	0.002	−1.225	0.221	−0.011	0.002
Working in this hospital since	→	Societal challenges	→	Emotion Focused	−0.004	0.003	−1.568	0.117	−0.011	−2.948 × 10^−4^
Current working/shift time	→	Personal life challenges	→	Emotion Focused	−2.569 × 10^−4^	9.749 × 10^−4^	−0.264	0.792	−0.006	0.002
Current working/shift time	→	Workplace challenges	→	Emotion Focused	0.005	0.003	1.718	0.086	7.773 × 10^−4^	0.013
Current working/shift time	→	Societal challenges	→	Emotion Focused	0.005	0.003	1.610	0.107	5.260 × 10^−5^	0.013
Distance to green spaces	→	Personal life challenges	→	Emotion Focused	−2.498 × 10^−4^	9.400 × 10^−4^	−0.266	0.790	−0.006	0.002
Distance to green spaces	→	Workplace challenges	→	Emotion Focused	8.377 × 10^−4^	0.002	0.467	0.641	−0.002	0.005
Distance to green spaces	→	Societal challenges	→	Emotion Focused	0.003	0.002	1.452	0.147	1.635 × 10^−4^	0.008
Education	→	Personal life challenges	→	Problem Focused	1.969 × 10^−4^	0.002	0.098	0.922	−0.006	0.010
Education	→	Workplace challenges	→	Problem Focused	−9.103 × 10^−4^	0.006	−0.153	0.878	−0.015	0.008
Education	→	Societal challenges	→	Problem Focused	0.002	0.002	0.683	0.495	−0.002	0.013
Current designation	→	Personal life challenges	→	Problem Focused	−1.178 × 10^−4^	0.001	−0.097	0.922	−0.008	0.004
Current designation	→	Workplace challenges	→	Problem Focused	0.008	0.007	1.189	0.234	−8.515 × 10^−4^	0.024
Current designation	→	Societal challenges	→	Problem Focused	−0.002	0.003	−0.797	0.425	−0.013	0.002
Working in this hospital since	→	Personal life challenges	→	Problem Focused	−1.523 × 10^−4^	0.002	−0.098	0.922	−0.006	0.006
Working in this hospital since	→	Workplace challenges	→	Problem Focused	−0.007	0.005	−1.264	0.206	−0.029	0.002
Working in this hospital since	→	Societal challenges	→	Problem Focused	0.005	0.005	1.014	0.310	−0.005	0.019
Current working/shift time	→	Personal life challenges	→	Problem Focused	−1.836 × 10^−4^	0.002	−0.098	0.922	−0.008	0.006
Current working/shift time	→	Workplace challenges	→	Problem Focused	0.012	0.006	1.831	0.067	0.001	0.031
Current working/shift time	→	Societal challenges	→	Problem Focused	−0.006	0.006	−1.026	0.305	−0.020	0.006
Distance to green spaces	→	Personal life challenges	→	Problem Focused	−1.786 × 10^−4^	0.002	−0.098	0.922	−0.008	0.005
Distance to green spaces	→	Workplace challenges	→	Problem Focused	0.002	0.004	0.469	0.639	−0.003	0.013
Distance to green spaces	→	Societal challenges	→	Problem Focused	−0.003	0.003	−0.981	0.327	−0.013	0.003
Education	→	Personal life challenges	→	Meaning focused	0.002	0.002	0.981	0.326	−8.275	0.009
Education	→	Workplace challenges	→	Meaning focused	−2.091	0.001	−0.152	0.879	−0.005	0.002
Education	→	Societal challenges	→	Meaning focused	−0.005	0.006	−0.879	0.379	−0.018	0.006
Current designation	→	Personal life challenges	→	Meaning focused	−0.001	0.002	−0.593	0.553	−0.007	0.001
Current designation	→	Workplace challenges	→	Meaning focused	0.002	0.002	0.878	0.380	−4.110	0.007
Current designation	→	Societal challenges	→	Meaning focused	0.008	0.007	1.178	0.239	−0.005	0.023
Working in this hospital since	→	Personal life challenges	→	Meaning focused	−0.002	0.002	−0.922	0.356	−0.008	0.001
Working in this hospital since	→	Workplace challenges	→	Meaning focused	−0.002	0.002	−0.907	0.364	−0.011	7.302
Working in this hospital since	→	Societal challenges	→	Meaning focused	−0.017	0.006	−2.894	0.004	−0.029	−0.006
Current working/shift time	→	Personal life challenges	→	Meaning focused	−0.002	0.002	−1.008	0.313	−0.008	6.576
Current working/shift time	→	Workplace challenges	→	Meaning focused	0.003	0.003	1.061	0.289	−6.783	0.010
Current working/shift time	→	Societal challenges	→	Meaning focused	0.020	0.006	3.191	0.001	0.010	0.035
Distance to green spaces	→	Personal life challenges	→	Meaning focused	−0.002	0.002	−1.161	0.246	−0.008	−4.125
Distance to green spaces	→	Workplace challenges	→	Meaning focused	4.556	0.001	0.441	0.659	−5.502	0.004
Distance to green spaces	→	Societal challenges	→	Meaning focused	0.010	0.005	2.311	0.021	0.002	0.021

*Note.* Delta method standard errors, bias-corrected percentile bootstrap confidence intervals, ML estimator.

**Table 3:** Total effects of demographics on coping.

							95% Confidence Interval	
			Estimate	Std. Error	z-Value	*p*	Lower	Upper
Education	→	Emotion Focused	−0.004	0.022	−0.186	0.852	−0.051	0.046
Current designation	→	Emotion Focused	0.045	0.025	1.812	0.070	−0.016	0.103
Working in this hospital since	→	Emotion Focused	0.048	0.019	2.567	0.010	0.012	0.081
Current working/shift time	→	Emotion Focused	0.114	0.020	5.707	<0.001	0.077	0.151
Distance to green spaces	→	Emotion Focused	0.062	0.016	3.931	<0.001	0.027	0.092
Education	→	Problem Focused	0.292	0.044	6.676	<0.001	0.214	0.376
Current designation	→	Problem Focused	0.184	0.048	3.807	<0.001	0.097	0.285
Working in this hospital since	→	Problem Focused	0.118	0.037	3.200	0.001	0.030	0.192
Current working/shift time	→	Problem Focused	0.203	0.039	5.175	<0.001	0.129	0.280
Distance to green spaces	→	Problem Focused	0.061	0.031	1.989	0.047	−0.005	0.126
Working in this hospital since	→	Meaning focused	0.097	0.024	4.013	<0.001	0.049	0.153
Current working/shift time	→	Meaning focused	0.055	0.025	2.229	0.026	0.008	0.098
Distance to green spaces	→	Meaning focused	0.018	0.020	0.894	0.371	−0.019	0.056

*Note.* Delta method standard errors, bias-corrected percentile bootstrap confidence intervals, ML estimator.

## Data Availability

Data presented in this study are available on request from the corresponding author.
